# SUPeRO: A Multidimensional Approach to Prevent and Manage Oncological Frailty in a Radiation Oncology Unit

**DOI:** 10.3390/jcm11226768

**Published:** 2022-11-15

**Authors:** Beatrice Di Capua, Marialuisa Iervolino, Alessandra Rocconi, Serena Bracci, Elisa Marconi, Loredana Dinapoli, Francesco Presta, Maria Antonietta Gambacorta, Luca Tagliaferri, Fabio Marazzi, Vincenzo Valentini, Giuseppe Ferdinando Colloca

**Affiliations:** 1 UOC di Radioterapia Oncologica, Dipartimento Diagnostica per Immagini, Radioterapia Oncologica ed Ematologia, Fondazione Policlinico Universitario Agostino Gemelli IRCCS, 00168 Roma, Italy; 2 Dipartimento di Scienze Geriatriche e Ortopediche, Università Cattolica del Sacro Cuore, 00168 Roma, Italy; 3 UOS di Psicologia Clinica, Fondazione Policlinico Universitario Agostino Gemelli IRCCS, 00168 Roma, Italy; 4 Dipartimento di Scienze Radiologiche ed Ematologiche, Università Cattolica del Sacro Cuore, 00168 Roma, Italy

**Keywords:** elderly, frailty, oncology, supportive care, geriatric assessment, geriatric oncology, personalized medicine

## Abstract

Currently, the management of older cancer patients is directed by a personalized approach and, where possible, a tailor-made treatment. Based on our previous experiences and considering the opportunity of combining a geriatric department and a radiation-oncology department, we have developed a path that follows the patient from the beginning of the treatment, taking into account the complications/late toxicities and the survivors. This study aimed to evaluate the impact of remodeling and restructuring some oncology, radiotherapy, and geriatrics services based on the primary evidence for managing older cancer patients. In 2020, Gemelli ART underwent 60,319 radiation-oncology treatments, admitted 943 patients in the radiation-oncology and supportive care ward, and treated and followed 15,268 patients in clinics. The average length of stay of the admitted patients was reduced from 20.6 days to 13.2 days. In 2021, 1196 patients were assessed for frailty, 847 were admitted for toxicity, and 349 patients were evaluated within the geriatric oncology and supportive care outpatient clinic, and it was found that 59.2% were fit, 31.6% were vulnerable, and 9.2% were frail. This experience has shown a reduction in hospitalizations and the average hospital stay of patients in the case of side effects, a high toxicity to treatments, and the possibility of treating patients with a high level of complexity. This approach should represent the future target of geriatric oncology with the global management of older or complex patients with cancer.

## 1. Introduction

The concept of multidisciplinary for the best management of cancer patients is becoming increasingly established in oncology. However, the presence of a multispecialty team can sometimes lead to a dispersion of energy and a missed benefit for the patient. In this scenario, it is well known that cancer is associated with aging, with incidence increasing with age, and the aging population is profoundly changing the oncology setting. Where, 20 years ago, age cut-offs for various cancer treatments were routine for older people with cancer, now weighing up the efficacy and tolerability of treatment needs to be individualized to provide a tangible response to a growing population of older fit patients. Geriatric oncology, originally conceived to help the oncologist assess and manage the older patient, now plays a much more fundamental role in guiding and helping cancer teams cope with numerous complex situations.

### 1.1. Frailty and Supportive Care of Complex Patients

Frailty is a clinical syndrome of physiological decline characterized by a significant vulnerability to adverse health outcomes, including hospitalizations, surgical complications, disability, dependency, and mortality [1,2,3]. An awareness of frailty and the associated risks for adverse health outcomes can improve the care for this most vulnerable subset of patients. Cancer can be the stressor that reveals the frail status of older patients [4]. For this purpose, the American Society of Clinical Oncology and the International Society for Geriatric Oncology (SIOG) recommend the routine use of a Comprehensive Geriatric Assessment (GCA) for older patients with cancer (≥70 years old) [5,6,7]. The SIOG recommends a variety of instruments that can be tailored to any patient [6]: performance-based evaluations such as the Timed Up and Go Test (TUAGT) [8], balance testing [9], grip strength [10], sit-to-stand test [11] and cognitive screening using the Clock Drawing Test [12], number of comorbidities, polypharmacy, socio-economic status and social network, caregiver presence, and distress [6]. Self-report measures are also commonly included in the CGA, such as the Geriatric Depression Scale (GDS) [13], Activities of Daily Living Scale [14], Instrumental Activities of Daily Living [15], quality-of-life measures [16], and a nutritional assessment [17]. Including both self-report and performance-based evaluations, the CGA provides the patient with a perception of functioning at home in conjunction with an objective assessment that may help providers develop realistic management plans [18]. In addition to the accurate and prompt identification of vulnerable and frail individuals through the CGA, appropriate supportive care is essential to support all patients during treatment, regardless of the degree of frailty. The SIOG and ASCO recommend carrying out at least one screening test when a CGA cannot be performed [6]. Currently, the most recommended and used is the G8 [19]. Supportive care in cancer is the prevention and management of the adverse effects of cancer and its treatments. This includes the management of physical and psychological symptoms and side effects from a patient’s diagnosis through their treatment, and to their post-treatment care [20]. Supportive care aims to maintain or improve one’s quality of life and maximize the benefits people can achieve from their anticancer treatments.

### 1.2. Multidimensional Approach for Oncological Frailty at Gemelli ART

Gemelli Advanced Radiation Therapy (Gemelli ART) is the new Cancer Radiation-Oncology Center at Fondazione Policlinico Universitario Agostino Gemelli IRCCS in Rome. Gemelli ART supports patients from their diagnosis to treatment through various services: outpatient facilities, clinics, radiotherapy, and an acute ward. The high level of innovation and the number of technologies active at Gemelli ART allow for a very high standard of care for diagnostic and therapeutic needs. The motto is “technology to serve knowledge, knowledge to serve patients”. Gemelli ART, since its birth, has enriched its team with multidisciplinary professionals, such as medical oncologists, psychologists, and physicists. In 2016, Gemelli ART started its collaboration with the geriatric department through the participation of a geriatrician who specialized in geriatric oncology in tumor boards and for the assessment and management of complex patients. In April 2017, a geriatrician was hired in the department, with his main activity being in the radiotherapy ward. In January 2020, a second geriatrician who specialized in geriatric oncology and supportive care was employed to extend the service. In October 2021, the multidisciplinary team of supportive therapies acquired a well-defined conformation with renovating the clinics and inpatient wards. The new ward has been renovated according to the principles of well-being and respect for frail oncology patients. It houses nine patients, including three in single rooms. There is an immersive room in the ward reserved for inpatients. The ward’s medical team consists of geriatricians, radiation oncologists, and psychologists operating as a team. When needed, the geriatrician can be called for an urgent consultation for patients followed in clinics or for outpatients undergoing radio-oncology treatment. The urgent consultation permits the decision of whether the patient needs to be hospitalized or can be followed with outpatient activity. This study aimed to evaluate the impact of the remodeling and restructuring of some oncology, radiotherapy, and geriatrics services based on the primary evidence for the management of older patients with cancer.

## 2. Material and Methods

The project has been named SUPeRO (Supportive in Radiation Oncology). It includes outpatient and inpatient services performed by the multidisciplinary team. The multidisciplinary team consists of three radiation oncologists; one oncologist, two geriatricians who specialize in geriatric oncology, a dermatologist, an endocrinologist, two psychologists, two rehabilitation physicians, anesthesiologists for pain therapy, and nurses. The outpatient services which are provided are a supportive therapy outpatient clinic for the management of toxicities, an oncogeriatrics outpatient clinic for the assessment and management of toxicity in frail patients, skin toxicity, and advanced wound medication, functional aesthetics in oncology, pain therapy, dysfunctional disorders, rehabilitation, and psychology (Table 1).

### Organization of Patient Flows

**Figure 1:**
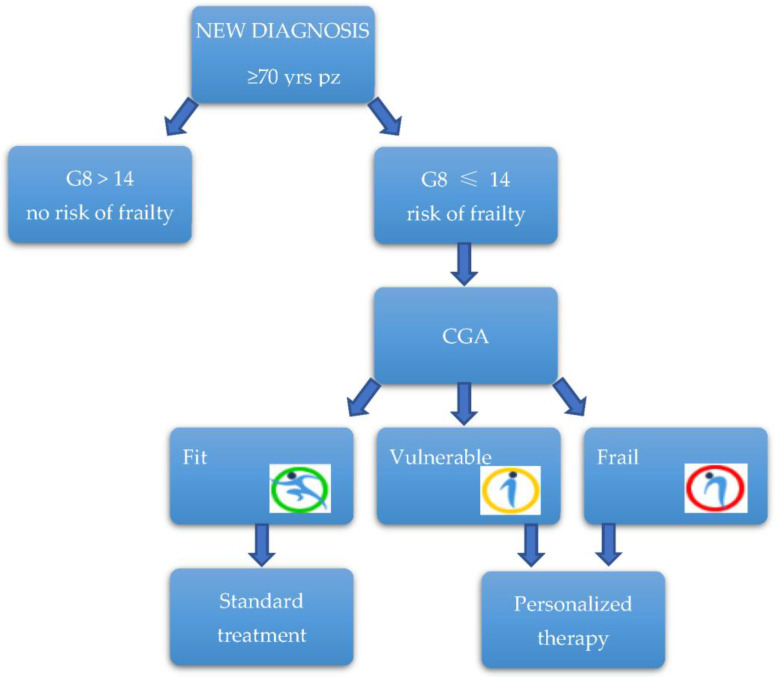
Patients flow.

All older patients (≥70 years old) admitted to the Gemelli ART were screened for frailty by the G8 screening tool: if the patient appeared not to be at risk, standard treatment will be offered; otherwise, if the patient is at risk of being frail, an oncogeriatric visit and a comprehensive geriatric assessment (CGA) were performed (Figure 1).

The CGA is composed of the evaluation of different domains through validated questionnaires and scales: it is focused on comorbidities (Cumulative Illness Rating Scale for Geriatrics), polypharmacotherapy (the number of drugs), the presence of geriatric syndromes, physical performance (Short Physical Performance Battery, Chair Stand test, walking speed, and handgrip strength), functional autonomy (Activity of Daily Living and Instrumental Activity of Daily Living), physical activity (Physical Activity Scale for Elderly), pain (Numerical Rating Scale or Visual Analogue Scale), quality of life (EuroQol 5D), mood and sleep disorders (Geriatric Depression Scale), the screening of cognitive decline (Mini-Mental State Examination and Clock Drawing Test or Montreal Cognitive Assessment), and a nutrition assessment (Mini Nutritional Assessment) (Table 2).

The interview investigates the social conditions and the presence of a caregiver, which are fundamental to programming an appropriate treatment for the patient. We also investigate any emerging symptoms that could affect the treatment compliance to manage it early. After the visit, the oncogeriatrician expresses a final opinion on whether the patient can be considered fit, vulnerable, or frail. In specific conditions, special indications referring to particularly frail areas of the patients can be given, such as red flags on the risk of malnutrition, sarcopenia, or delirium. By the CGA, the geriatrician evaluates if the patient can be considered frail, vulnerable, or fit. In the first two cases, a personalized treatment based on the frailty area of the patient will be discussed among the multidisciplinary team. A tailor-made treatment was performed using all the services explained above, with a continuous exchange between the radiotherapist and the geriatrician on how the treatment is proceeding. Unfit or frail patients are followed during and after the treatment to monitor their tolerance to the treatment.

## 3. Results

**Figure 2:**
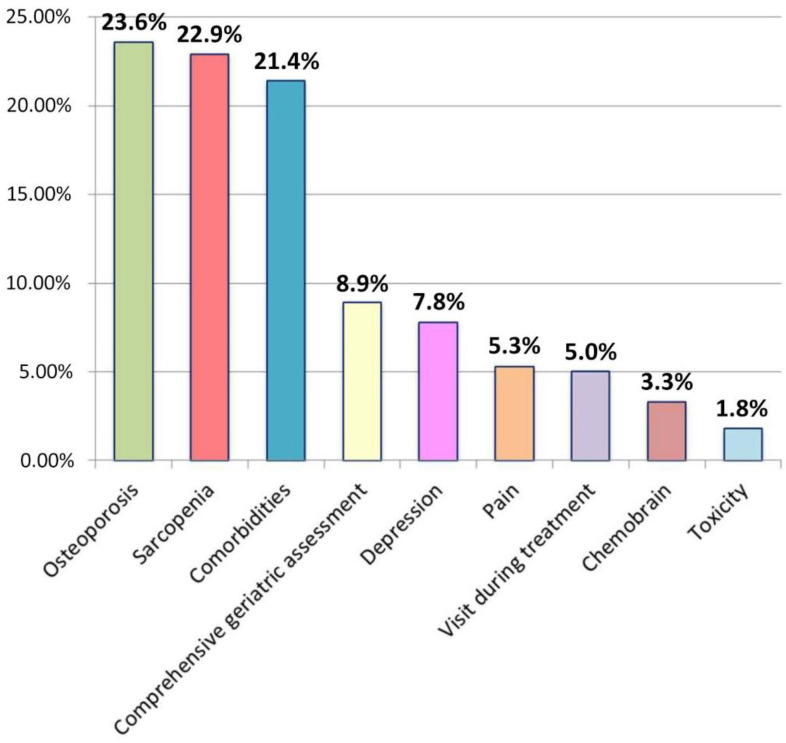
Reasons for outpatient evaluations.

**Figure 3:**
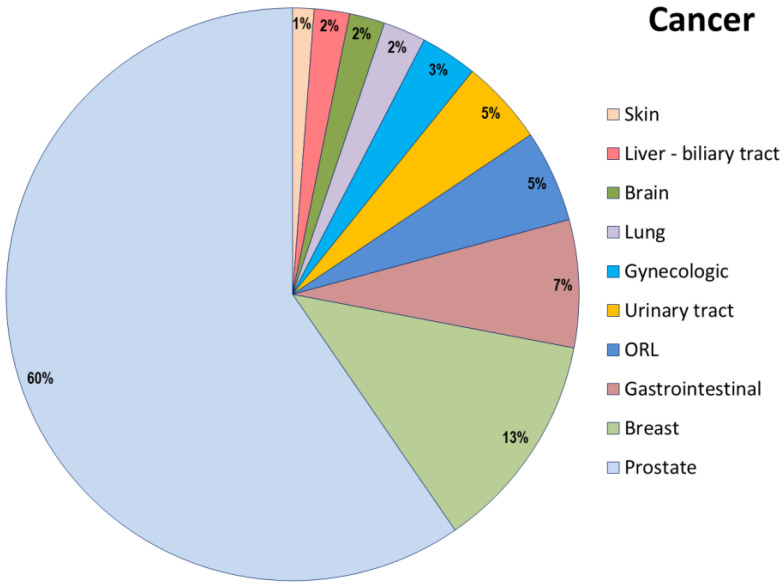
Cancer type in the outpatient population.

In 2020, Gemelli ART underwent 60,319 radiation-oncology treatments, admitted 943 patients in the radiation-oncology and supportive care ward, and treated and followed 15,268 patients in clinics. We observed an increase in the number of older patients treated with standard treatments and in the number of patients admitted, with a reduction in the average length of stay of these patients from 20.6 days in 2016 (last year before the restructuring and reorganization of the units) to 13.2 days. In 2021, 1196 patients were assessed for their frailty before starting radiation-oncology treatments, 847 were admitted for toxicity or to undergo oncological treatments, 349 patients were evaluated within the geriatric oncology and supportive care outpatient clinic, for a total of 597 visits (258 first contact and 339 follow-ups). The mean age was 75 years; all patients aged ≥ 70 were assessed by a G8 screening test, and patients at risk of being frail at G8 were assessed by a comprehensive geriatric assessment, and it was found that 59.2% were fit, 31.6% were vulnerable, and 9.2% were frail, following Balducci’s criteria [21]. The reasons for outpatient evaluations are shown in Figure 2. The cancer type is shown in Figure 3.

## 4. Discussion

The Radiation-oncology and Supportive Care Unit is a unique entity compared to usual hospitals worldwide. Its activities include supportive care during treatments, the management of toxicities in patients undergoing an oncology treatment, and performing radiotherapy or chemotherapy treatment in frail or complex patients. The presence of a geriatrician who specializes in geriatric oncology permits us to perform oncological therapies for older patients in a protected condition. For frail or vulnerable patients, concomitant chemotherapy is preferentially administered during a short hospitalization that allows us to control the possible side effects and sustains the patients with supportive care. The patients are also admitted for toxicity management or complications of tumor progression from the emergency department or their home, both with a preferential track. Primary admission diagnoses are sepsis and other infections, mucositis, respiratory failure, renal and liver failure, neurological issues, pain, dysphagia, and malnutrition. The early resolution of these complications permits a restarting of the oncological treatment quickly, benefiting a patients’ quality of life and survival. Since a geriatrician started working in the ward, we observed an increase in the number of older patients admitted and in the number of patients admitted from the emergency department, together with a reduction in the average length of stay of these patients from 20.6 days in 2016 to 13.2 days.

## 5. Conclusions

Following the scientific evidence and guidelines that highlight how frailty is a dynamic and heterogeneous concept, it is advisable to conduct its evaluation not only before the treatments but also during and after the treatments. This study highlighted how by reshaping the services and departments of a Cancer Center based on evidence for the best management of older patients with cancer, it is possible to improve patient management not only in qualitative terms (for example, their quality of life and performance) but also quantitative (for example, the average length of the hospital stay or a reduction in the toxicity to treatments). The multidimensional and multidisciplinary approach through multiple services allows the patient to be followed during the course of their care accurately and proportionately to their needs, with the aim of completing the planned treatment, reducing the G3–G4 toxicity rate, and, in general, the emergency department admissions.

This work represents a new methodology in following older patients with cancer along their diagnostic and therapeutic path and consequently represents an overcoming of the tailor-made treatment that should be developed for the patient.

## Figures and Tables

**Table 1:** Outpatient services performed by the multidisciplinary team.

Clinics	
Geriatric oncology	Comprehensive Geriatric Assessment.
	Management of frail patients.
	Management of osteoporosis and sarcopenia.
Supportive care	Management of toxicities during treatments (fatigue, nausea, vomiting, dyspepsia, constipation, diarrhea, fecal and urinary incontinence, dysphagia, xerostomia, and neurological disorders).
Pain	Multidisciplinary management of cancer pain.
Tegument toxicity	Multidisciplinary management of skin and mucosal toxicities during radiotherapy or chemotherapy.
Functional aesthetics in oncology	Treatment of skin blemishes during radiation therapy.
Advanced wound medication	Daily nursing dressing.
Psycho-oncology	Psychological support during treatment.
Endocrine and sexual dysfunction	Specialist management of endocrinological and sexual dysfunction during radiotherapy or hormone therapy.
Rehabilitation	Evaluation and prescription of rehabilitation before, during, and after cancer treatments.

**Table 2:** The comprehensive geriatric assessment used in our center.

Domain	Tools
Comorbidities Polypharmacy	Cumulative Illness Rating Scale for Geriatrics (CIRS-G).
	Number of drugs.
	Geriatric Syndromes.
Physical performance	Short Physical Performance Battery (SPPB).
	Chair Stand test.
	Walking speed.
	Handgrip.
Functional performance	The activity of Daily Living (ADL).
	Instrumental Activity of Daily Living (IADL).
Physical activity	Physical Activity Scale for the Elderly (PASE).
Pain	Numerical Rating Scale (NRS).
	Visual Analogue Scale (VAS).
Quality of life	EuroQol 5D.
Mood and sleep disorders	Geriatric Depression Scale (GDS).
Cognitive impairment	Mini-Mental State Examination (MMSE).
	Clock Drawing Test.
	Montreal Cognitive Assessment (MOCA).
Nutrition	Mini Nutritional Assessment (MNA).

## Data Availability

Not applicable.
